# Study of the Navigation Method for a Snake Robot Based on the Kinematics Model with MEMS IMU

**DOI:** 10.3390/s18030879

**Published:** 2018-03-16

**Authors:** Xu Zhao, Lihua Dou, Zhong Su, Ning Liu

**Affiliations:** 1 School of Automation, Beijing Institute of Technology, Beijing 100081, China; zhaoxu00@126.com (X.Z.); sz@bistu.edu.cn (Z.S.); 2 Beijing Key Laboratory of High Dynamic Navigation Technology, Beijing Information Science & Technological University, Beijing 100101, China; ning.liu@bistu.edu.cn

**Keywords:** snake robot, MEMS IMU, inertial navigation system mechanization, kinematics model

## Abstract

A snake robot is a type of highly redundant mobile robot that significantly differs from a tracked robot, wheeled robot and legged robot. To address the issue of a snake robot performing self-localization in the application environment without assistant orientation, an autonomous navigation method is proposed based on the snake robot’s motion characteristic constraints. The method realized the autonomous navigation of the snake robot with non-nodes and an external assistant using its own Micro-Electromechanical-Systems (MEMS) Inertial-Measurement-Unit (IMU). First, it studies the snake robot’s motion characteristics, builds the kinematics model, and then analyses the motion constraint characteristics and motion error propagation properties. Second, it explores the snake robot’s navigation layout, proposes a constraint criterion and the fixed relationship, and makes zero-state constraints based on the motion features and control modes of a snake robot. Finally, it realizes autonomous navigation positioning based on the Extended-Kalman-Filter (EKF) position estimation method under the constraints of its motion characteristics. With the self-developed snake robot, the test verifies the proposed method, and the position error is less than 5% of Total-Traveled-Distance (TDD). In a short-distance environment, this method is able to meet the requirements of a snake robot in order to perform autonomous navigation and positioning in traditional applications and can be extended to other familiar multi-link robots.

## 1. Introduction

The snake robot, which is based on the biological characteristics of snakes, constitutes an important branch of bionic robots. Prof. Hirose, S. developed the first snake robot in 1972 [1]. A snake robot is significantly different from a tracked robot, wheeled robot and legged robot, being a mobile robot with high redundancy. Because of the multi-joint flexible structure design, a snake robot has the advantage of multi-gait motion and the ability to adapt to a complex unknown environment, and can be widely used in disaster rescue, underwater survey, industrial testing and other special environments that traditional robots or humans cannot enter; as a result, increasing attention is being paid to snake robots [2,3].

In 1946, Gray, J., at Cambridge University, divided movement gaits into serpentine movement, rectilinear movement, concertina movement and sidewinding movement in the study of the biological nature of snakes [4]. Shigeo Hirose at Tokyo University established a serpentine gait kinematics model with linkage structures based on the observation of biological snake movement processes and bone anatomy [1]. Liljebäck, P. et al. at the Norwegian University, analyzed the position relationship between a snake robot and obstacles, proposed an obstacle assistant movement gait in planar motion, and built the kinematics and dynamics model for the snake robot [5,6]. In studying the snake robot’s body, the team of Hirose, S. at Tokyo University developed a series of ACM snake robots, in which the ACM-R5 snake robot had amphibious motion ability [7]. A Carnegie Mellon University team studied a unified Snake robot with climbing ability [8]. The Norwegian Technology University developed the snake robot Anna Konda [9] for fire rescue and developed the snake robot Kulko [10] with stress perception.

The Snake robot’s autonomous navigation and positioning is the premise of the autonomous movement in complex unknown environments [3]. At present, most research is related to the snake robot’s structure, the movement gait, and the gait control method and robot body; however, most of these robots do not have the ability to perform autonomous navigation and positioning, which has hindered their use in complex unknown environments [2]. The only relevant work includes: Tanaka, M. et al., who developed a set of half self-help collision avoidance systems for a snake robot using LiDAR and realized the robot’s autonomous motion in a limited space [11]; Tian, Y. et al. who proposed a SLAM algorithm using single LiDAR, with LiDAR fixed on the outside of the robot head joints, and generated a 2D environment map for navigation [12]; and Chavan, P., who realized mapping and navigation for a snake robot using an Ultrasonic-sensor and a PIR-sensor, along with wireless control through ZigBee [13]. Some sensors, such as LiDAR and Ultrasonic, can measure environment depth information, which can be used in a snake robot’s navigation and positioning. However, these sensors have too large a volume and require too much energy to be used in a snake robot system. Fu, X. et al. proposed a snake robot control system based on a visual tracking algorithm and realized tracking ground path navigation using a monocular camera [14]. Xiao, X. et al. used two external cameras to detect a snake robot’s posture and simplified the attitude estimation by putting fiducials on the snake robot [15]. Visual navigation has high requirements on light, which limits its use of the environment, and often tends to require a high computational effort, increasing the cost of the system. Ponte, H. et al. designed a triangulation measurement sensor that adapts to the size and power of the snake robot using a laser and a black and white camera; the robot can scan an environmental color 3D point cloud when the head is raised, with the robot’s posture estimated using the kinematics equation and IMU data [16]. Ohno, K. et al. combined the information from an IMU and a TOF camera, performed estimation of the snake robot’s trajectory, and completed 3D SLAM reconstruction [17]. A structured light sensor or a TOF camera combined with IMU information can realize the reconstruction of the environmental depth and robot’s navigation and positioning; however, the algorithm used is complex, and the number of calculations is high. Billah, M.M. et al. proposed a navigation system for a snake robot using intelligent inertial sensors to well overcome the limits of Wi-Fi, RFID and other positioning systems; however, the paper did not mention the positioning accuracy [18]. Estébanez, J.G. designed a navigation system for snake robots using integrated GPS and IMU, and developed a kinematic physical model which simulated the robot motion and estimated the robot trajectory when the GPS signal reception was not able to [19]. Yang, W. et al. developed a type of snake robot motion-tracking system with a low-cost MEMS IMU that used three algorithms (low-pass filter, baseline calibration and Kalman filter) to remove noise from the IMU acceleration data and evaluated the tracking algorithm’s efficiency using video tracking software; its maximum errors of velocity and position were 10.93% and 12.23% TTD, respectively. However, the system only considered the accelerometer data, which can only track linear motion [20]. In general, LiDAR/Ultrasonic/Visual/Structured light/TOF camera sensors tend to be bulky, highly energy consuming, or require high computational effort. The MEMS inertial sensor not only has a small size and low energy consumption, but is also low in computational load; this paper is based on the work of this sensor.

By analyzing the research work of scholars in the robot autonomous navigation positioning field, it can be seen that there is great difficulty in effectively achieving autonomous positioning, especially when the snake robot implements positioning without an external assistant in complex unknown environments. Inspired by the dead reckoning method for people [21], the main contribution of the paper is the proposition of an autonomous navigation positioning method based on the constraints of the snake robot’s motion characteristics, using the robot’s own MEMS IMU to realize the snake robot’s autonomous navigation and positioning with non-nodes (used to differentiate from similar Wi-Fi/ZigBee/Beacons and other navigation systems that need nodes and external assistant) and a non-external assistant. The system has small volume, low power consumption, and only requires a simple installation and a small amount of calculation; thus, the system can meet the requirement of the snake rescue robot’s autonomous navigation and positioning, and can be extended to other similar robot fields.

## 2. Analysis of the Snake Robot’s Motion Characteristics

**Figure 1:**
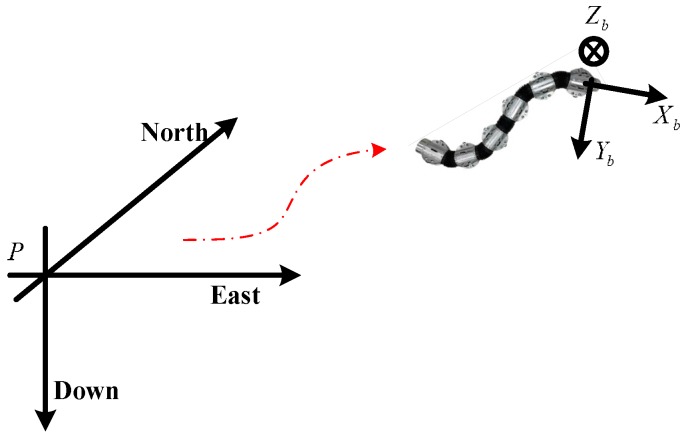
Navigation coordinate system of the snake robot.

A snake robot has multiple movement gaits, and the serpentine movement is the most studied and most efficient of all two-dimensional gaits. It transfers with a lateral wave, and the motion curve is similar to a sine curve. The phase and amplitude fluctuations change over time. The snake robot navigation algorithms in this paper are based on the study of the aforementioned gait. The navigation coordinate system (*n* coordinate) defines the origin as the navigation system’s point *P*, with axes pointing north, east and the local vertical direction (down); the Carrier coordinate system (*b* coordinate) is shown in Figure 1; the Carrier coordinate system and the snake robot body are fixed, with axes pointing to the front, right and bottom of the snake robot’s movement direction.

### 2.1. Snake Robot Kinematics Model

The serpentine curve was first proposed by Hirose [1]; Ma et al. proved that the curve had the best movement efficiency from continuous muscle contraction [22]. The serpentine movement Serpenoid curve’s curvature equation is expressed as [23]:

(1) ρ=−αbsin(bs)

where *ρ* is the curvature of the curve, *α* is the initial amplitude angle of curve, *b* is the adjustable constant, and *s* is the length of curve.

**Figure 2:**
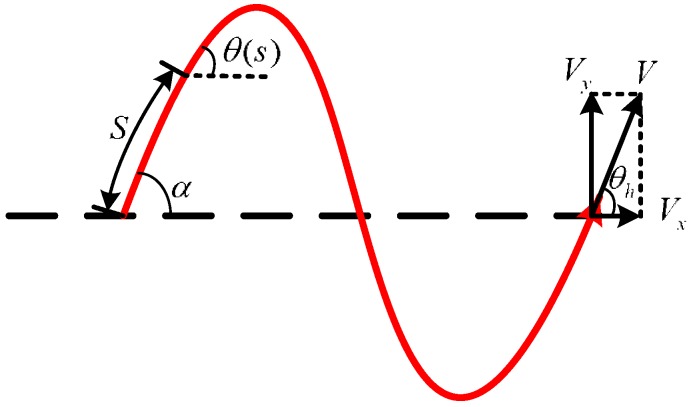
Serpenoid curve.

**Figure 3:**
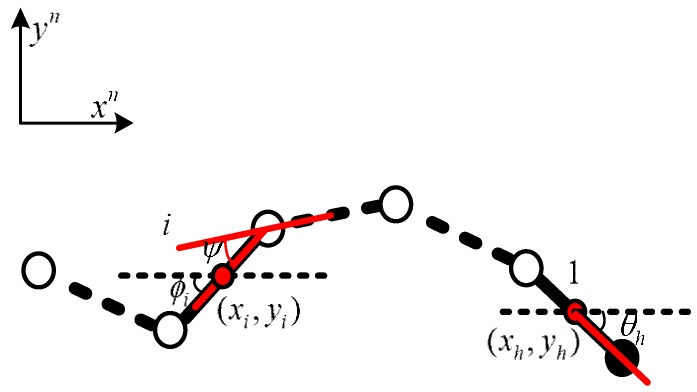
Relative joint angle.

We assume that the robot consists of *N* joints of length 2*l*, and the joints are connected by *N* − 1 motors. Every joint has the same mass m except for the head joint; the mass of each joint is uniformly distributed, and the center of mass is located at the center point (i.e., at a distance *l* from the two ends of the joint). Neglecting the width of the snake robot, the robot’s movement is shown in Figure 2. The snake robot head mass is *m_h_*, IMU is in the head of the centroid position. As shown in Figure 3, $x_{h},y_{h}$ corresponds to the coordinate position for the snake robot head at a certain moment, $x_{i},y_{i}$ to the coordinate position for the joint *i.* The definition of *S* facilitates the calculation of the angle between the relative motion directions of robot joints, whereby joint *i* corresponds to S=i×2l−1. This angle is represented by θ(s) in Figure 2 and can be expressed as:

(2) θ(s)=αcos(bs)

The angle between the joints shown in Figure 3 can be expressed as:

(3) ψ=θ(s+l)−θ(s−l)=−2αsin(bl)sin(bs)

According to the serpentine curve and variable *S* (the length of the snake robot), we can obtain the input angle formula of each joint (as shown in Figure 2 and Figure 3: *θ_h_* is the angle between the head and the motion direction, and $\phi_{i}$ is the angle between joint *i* and the motion direction):

(4) $$\begin{array}{c} \theta_{h}(t)=A\sin(\omega t-0.5\vartheta )\\ \phi_{i}(t)=A\sin(\omega t+(i-1)\vartheta ) \end{array}$$

where *A* is the amplitude, A=−2αsin(bl), bs=ωt, ω is the angular velocity, ϑ is the phase, i=1:N, and *N* is the total number of joints. Angular changes between adjacent joints drive the snake robot’s serpentine movement. As shown in Figure 3, the direct geometric relationship expression of each joint position is expressed as:

(5) $$\begin{array}{c} x_{i}=x_{h}+2l\cos(\theta_{h})+2l\sum_{k=1}^{i-1}\cos(\theta_{h}+\sum_{j=1}^{k}\phi_{j})+l\cos(\theta_{h}+\sum_{k=1}^{i}\phi_{k})\\ y_{i}=y_{h}+2l\sin(\theta_{h})+2l\sum_{k=1}^{i-1}\sin(\theta_{h}+\sum_{j=1}^{k}\phi_{j})+l\sin(\theta_{h}+\sum_{k=1}^{i}\phi_{k}) \end{array}\}$$

The initial geometry center position of the snake robot’s head is the origin. We set the global coordinate frame as the position of the snake robot’s head and the movement direction as the *x*-axis, with the *y*-axis being vertical to the *x*-axis.

The input joint angle can be deduced from Formula (5) and is used to control the snake robot’s movement. The method of changing the angle function to control the snake robot’s motion path is called the center adjustment method [24]. The mathematical expression is:

(6) $$\begin{array}{c} \theta_{h}(t)=A\sin(\omega t-0.5\vartheta )+\gamma_{h}\\ \phi_{i}(t)=A\sin(\omega t+(i-1)\vartheta )+\gamma_{i} \end{array}\}$$

If the input joint angle changes γ, the input angle of symmetry axis will deviate from the desired location (zero position) value γ. In this manner, the direction of the snake robot is changed to move toward the navigation points. γ is called the adjustment factor of direction.

### 2.2. Analysis of the Snake Robot’s Motion Restraint Characteristics

Khatib first applied the artificial potential field (APF) method in path planning guidance [25]. The basic principle is that obstacles have repulsion, targets have gravitational pull. An obstacle is in the area of high potential energy, releasing energy outward. A target is in the region of lower potential energy, absorbing the external energy. The potential field force can be represented as:

(7) $$E_{T}=k\times d^{2}$$

where *k* is a persistent target point, and *d* is the distance from the robot’s head to the target point.

We assume that the robot’s target environment is static, and the target coordinates are known. In the environment, the snake robot is affected by the obstacles’ repulsive forces and the targets’ attractive forces. The paper only considers the attractive forces’ influence and assumes that:

(8) $$F(t)=p\times E_{T}(t)$$

where *F(t)* is the attractive force, and *p* is a constant related to the robot.

**Figure 4:**
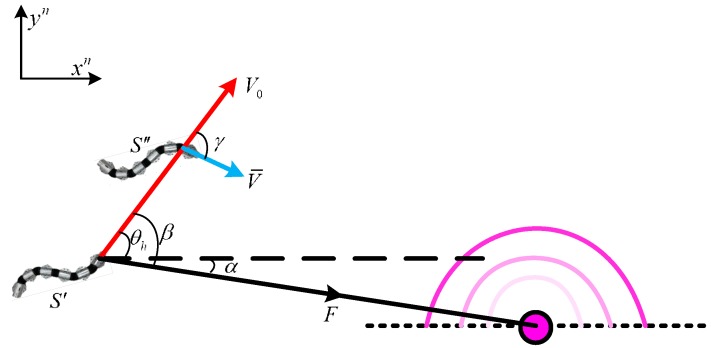
Relationship between the distance and the attractive force.

As shown in Figure 4, when the snake robot is on $S^{\prime }$, it is affected by the attractive force from the target and turns an angle γ. The velocity of snake robot’s head is $v_{0}$, the angle from the *x*-axis is $\theta_{h}$, the attractive force from the target to the robot on $S^{\prime }$ is *F*, the angle from the *x*-axis is α, and β is the angle between the attractive force *F* and velocity $v_{0}$.

**Figure 5:**
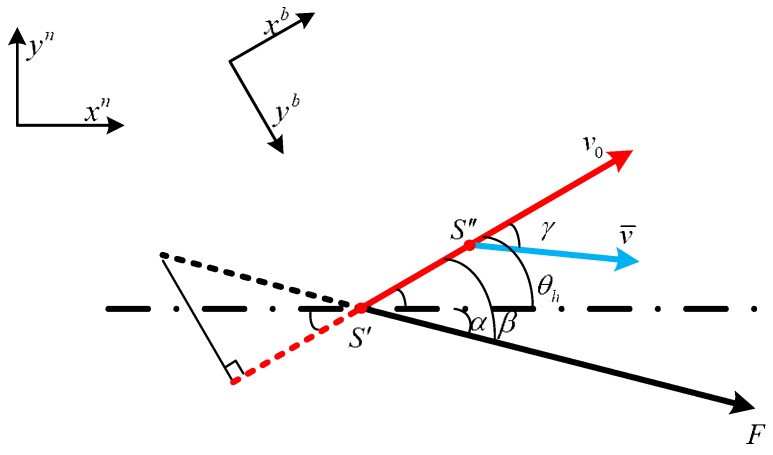
Relationship between the Force and the Velocity.

With no attractive force, the robot’s movement velocity in a short period dt is $v_{0}$. Under an attractive force, the velocity will become $\bar{v}$, and the corresponding velocity $v_{0}$ will change angle γ to move toward the target. To calculate the change angle γ, the relationship between force and velocity is shown in Figure 5:

To determine γ, the attractive force *F* is decomposed into two directions: one is parallel to $v_{0}$, and the other is vertical to $v_{0}$. Within a short time dt, the snake robot is under a uniformly accelerated process in both directions. According to Newton’s second law, we obtain:

(9) $$\begin{array}{c} v_{x^{b}}=v_{0}+F\sin(\beta )dt/m_{h}\\ v_{y^{b}}=F\cos(\beta )dt/m_{h}\\ \gamma =\arctan(v_{x^{b}}/v_{y^{b}})\\ \bar{v}=\sqrt{v_{x^{b}}{}^{2}+v_{y^{b}}{}^{2}} \end{array}\}$$

where $m_{h}$ is the mass of the robot’s head, $v_{x^{b}}$ is the component along the $x^{b}$ axis, and $v_{y^{b}}$ is the component along the $y^{b}$ axis.

(10) $$\begin{array}{c} \gamma =\arctan((v_{0}m_{h}+F\sin(\beta )dt)/F\cos(\beta )dt)\\ \bar{v}=\sqrt{m_{h}{}^{2}v_{0}{}^{2}+{\left(Fdt\right)}^{2}+2m_{h}v_{0}F\cos(\beta )dt}/m_{h} \end{array}\}$$

Substituting Formula (10) into Formula (6), we obtain the relationship between the direction adjustment function of the snake robot and the potential force *F*.

Formula (10) can be extended to multi-targets:

(11) $$\begin{array}{c} \gamma =\arctan((v_{0}m_{h}+\sum_{j=1}^{n}F_{j}\sin(\beta )dt)/\sum_{j=1}^{n}F_{j}\cos(\beta )dt)\\ \bar{v}=\sqrt{m_{h}{}^{2}v_{0}{}^{2}+{\left(\sum_{j=1}^{n}F_{j}dt\right)}^{2}+2m_{h}v_{0}\sum_{j=1}^{n}F_{j}\cos(\beta )dt}/m_{h} \end{array}\}$$

where *n* is the number of targets.

The influence of the target attractive force field *F* on the snake robot’s motion can be obtained by substituting Formula (10) into Formula (6). By adjusting γ, the robot can be controlled to avoid a collision. The snake robot’s head motion adjusting parameters are as shown in (12) [26]:

(12) $$\theta_{h}(t)=A\sin(\omega t-0.5\vartheta )+\arctan((v_{0}m_{h}+F\sin(\beta )dt)/F\cos(\beta )dt$$

From Formula (12) the potential field attractive force of target *F* can change the snake robot’s head motion direction $\theta_{h}(t)$ and movement speed. In practice, the snake robot calculates the direction to the target according to its speed, posture and position information. It is controlled to move in the correct direction by adjusting steering force $F_{k}$ to simulate the attractive force from the potential field. Although this method has been applied in the field of vehicle and pedestrian navigation for a long time, it has not been applied to snake robots because of the uniqueness of the movement characteristics of snake robots. The novelty of this paper is in applying this technology to a snake robot’s navigation by studying the motion model of the snake robot and using the above models, the snake robot motion controller can be designed to realize the robot’s multi-gait flexible movements.

### 2.3. Error Propagation Properties

Formula (12) contains the relationship between the control force *F_k_* and the angle that must be adjusted by the controller to realize the movement of the snake robot. In space, the snake robot’s own coordinate system is defined as the carrier coordinate system using superscript *b.* The navigation coordinate system is defined as the front right down coordinate system along the direction of the robot’s head direction with superscript *n*. These coordinate systems are shown in Figure 1. The snake robot’s movement process in the space can be simplified as follows:

(13) $$\left\{ \begin{array}{l} v^{n}\left(t\right)=\int_{t_{0}}^{t}a^{n}\left(t\right)dt+v^{n}\left(t_{0}\right)\\ r^{n}\left(t\right)=\int_{t_{0}}^{t}v^{n}\left(t\right)dt+r^{n}\left(t_{0}\right) \end{array}\right.$$

where v(t) is the snake robot motion vector speed at moment *t*, and r(t) is its motion vector position at moment *t*. In the process of the snake robot’s movement, its error of velocity, attitude and angle can be described as follows:

(14) $$\left\{ \begin{array}{l} \delta \varphi_{k+1}=\delta \varphi_{k}+C_{b_{k|k-1}}^{n}\delta \omega_{k}^{b}\Delta t+w_{\varphi_{k}}\\ \delta v_{k+1}=\delta v_{k}+C_{b_{k|k-1}}^{n}\delta a_{k}^{b}\Delta t+w_{v_{k}}\\ \delta r_{k+1}=\delta r_{k}+\delta v_{k}\Delta t+w_{r_{k}} \end{array}\right.$$

where $\delta \varphi_{k}$ is the attitude angle error at the time *k* and is defined as a column vector, containing the roll angle, pitching angle and yaw angle. $C_{b_{k|k-1}}^{n}$ is the direction cosine matrix at the time *k*, the details of which will be presented in the next section. $\delta \omega_{k}^{b}$ is the bias of the gyro’s output. $\delta v_{k}$ is the three-axis velocity under the navigation system. $\delta r_{k}$ is the three-axis position under the navigation system. $\delta a_{k}^{b}$ is the bias of the accelerometer’s output. $w_{•}$ is the corresponding system error.

## 3. Navigation Method under the Constraint of the Snake Robot’s Motion Features

### 3.1. Mechanical Arrangement of the Strapdown Navigation System

**Figure 6:**
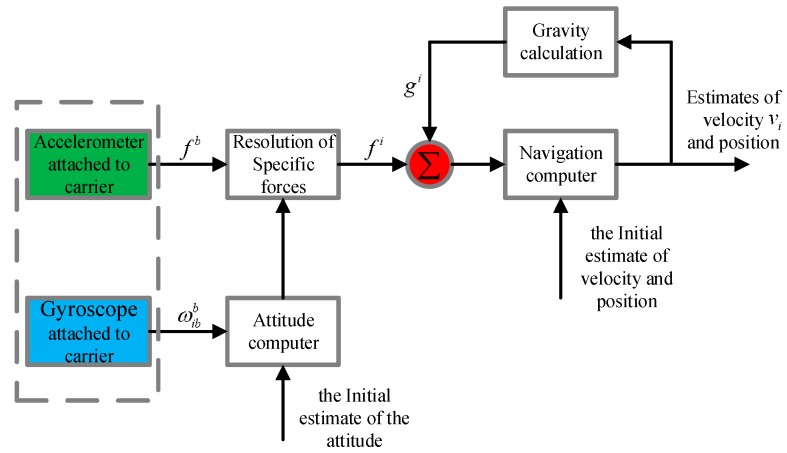
Arrangement of the machinery of the strapdown inertial navigation system.

The concept of the snake robot using the MEMS inertial measurement unit for navigation and positioning originally comes from the traditional robot’s navigation system design. The snake robot’s mechanical arrangement of the strapdown navigation system using MEMS-IMU in this paper is shown in Figure 6 [27]. Acceleration and angular velocity values of the carrier are measured separately by an accelerometer and gyroscope attached to a snake robot. The attitude and position of the carrier are calculated by the navigation computer through attitude calculation and force-resolution.

The snake robot’s navigation system can complete the initial alignment in 10 s after power on. According to the angular rate information, it updates the snake robot’s posture using the quaternion method. The quaternion update algorithm is as follows, calculating the angular increment first:

(15) $$\Delta =\sqrt{{\left(\omega_{x}T_{m}\right)}^{2}+{\left(\omega_{y}T_{m}\right)}^{2}+{\left(\omega_{z}T_{m}\right)}^{2}}$$

where Δ is the angular increment, $\omega_{x},\omega_{y},\omega_{z}$ is the three-axis angular velocity scalar value under the carrier system. $T_{m}$ is the sampling time. Next, the quaternion is updated:

(16) $$\left\{ \begin{array}{l} q_{1|k+1}=(1-\frac{\Delta^{2}}{8}+\frac{\Delta^{4}}{384})q_{1|k}-\omega_{x}T_{m}(0.5-\frac{\Delta^{2}}{48})q_{2|k}-\omega_{y}T_{m}(0.5-\frac{\Delta^{2}}{48})q_{3|k}-\omega_{z}T_{m}(0.5-\frac{\Delta^{2}}{48})q_{4|k}\\ q_{2|k+1}=(1-\frac{\Delta^{2}}{8}+\frac{\Delta^{4}}{384})q_{2|k}+\omega_{x}T_{m}(0.5-\frac{\Delta^{2}}{48})q_{1|k}+\omega_{z}T_{m}(0.5-\frac{\Delta^{2}}{48})q_{3|k}-\omega_{y}T_{m}(0.5-\frac{\Delta^{2}}{48})q_{4|k}\\ q_{3|k+1}=(1-\frac{\Delta^{2}}{8}+\frac{\Delta^{4}}{384})q_{3|k}+\omega_{y}T_{m}(0.5-\frac{\Delta^{2}}{48})q_{1|k}-\omega_{z}T_{m}(0.5-\frac{\Delta^{2}}{48})q_{2|k}+\omega_{x}T_{m}(0.5-\frac{\Delta^{2}}{48})q_{4|k}\\ q_{4|k+1}=(1-\frac{\Delta^{2}}{8}+\frac{\Delta^{4}}{384})q_{4|k}-\omega_{x}T_{m}(0.5-\frac{\Delta^{2}}{48})q_{3|k}+\omega_{y}T_{m}(0.5-\frac{\Delta^{2}}{48})q_{2|k}-\omega_{z}T_{m}(0.5-\frac{\Delta^{2}}{48})q_{1|k} \end{array}\right.$$

where $q_{1|k+1}$ is the first value of quaternion at time *k* + 1 and so on; $q_{1|k}$ is the first value of quaternion at time *k*. Next, normalize its quaternion:

(17) $$A=\sqrt{q_{1|k+1}{}^{2}+q_{2|k+1}{}^{2}+q_{3|k+1}{}^{2}+q_{4|k+1}{}^{2}}$$

(18) $$\left\{ \begin{array}{l} q_{1|k+1}^{\prime }=\frac{q_{1|k+1}}{A}\\ q_{2|k+1}^{\prime }=\frac{q_{2|k+1}}{A}\\ q_{3|k+1}^{\prime }=\frac{q_{3|k+1}}{A}\\ q_{4|k+1}^{\prime }=\frac{q_{4|k+1}}{A} \end{array}\right.$$

where *A* is the square sum of the quaternion at time *k* + 1. ${q^{\prime }}_{1|k+1}$ is the first normalized value of the quaternion at time *k* + 1, and so on. Next, the direction cosine matrix is obtained as follows:

(19) $$C_{b}^{n}=[\begin{array}{ccc} {\left(q_{1|k+1}^{\prime }\right)}^{2}+{\left(q_{2|k+1}^{\prime }\right)}^{2}-{\left(q_{3|k+1}^{\prime }\right)}^{2}-{\left(q_{4|k+1}^{\prime }\right)}^{2} & 2(q_{2|k+1}^{\prime }q_{3|k+1}^{\prime }-q_{1|k+1}^{\prime }q_{4|k+1}^{\prime }) & 2(q_{2|k+1}^{\prime }q_{4|k+1}^{\prime }+q_{1|k+1}^{\prime }q_{3|k+1}^{\prime })\\ 2(q_{2|k+1}^{\prime }q_{3|k+1}^{\prime }+q_{1|k+1}^{\prime }q_{4|k+1}^{\prime }) & {\left(q_{1|k+1}^{\prime }\right)}^{2}-{\left(q_{2|k+1}^{\prime }\right)}^{2}+{\left(q_{3|k+1}^{\prime }\right)}^{2}-{\left(q_{4|k+1}^{\prime }\right)}^{2} & 2(q_{3|k+1}^{\prime }q_{4|k+1}^{\prime }-q_{1|k+1}^{\prime }q_{2|k+1}^{\prime })\\ 2(q_{2|k+1}^{\prime }q_{4|k+1}^{\prime }-q_{1|k+1}^{\prime }q_{3|k+1}^{\prime }) & 2(q_{3|k+1}^{\prime }q_{4|k+1}^{\prime }+q_{1|k+1}^{\prime }q_{2|k+1}^{\prime }) & {\left(q_{1|k+1}^{\prime }\right)}^{2}-{\left(q_{2|k+1}^{\prime }\right)}^{2}-{\left(q_{3|k+1}^{\prime }\right)}^{2}+{\left(q_{4|k+1}^{\prime }\right)}^{2} \end{array}]$$

Next, we can obtain the corresponding attitude information:

(20) $$\{\begin{array}{c} \psi =\arctan\left(\frac{c_{32}}{c_{33}}\right)\\ \theta =\arcsin\left(-c_{31}\right)\\ \gamma =\arctan\left(\frac{c_{21}}{c_{11}}\right) \end{array}$$

Compensating the gravity according to the above matrix information and specific information, we can obtain the acceleration of the navigation frame and compute the velocity and position information at the same time.

(21) $${\overset{⌣}{a}}_{k}=C_{b_{k|k-1}}^{n}\cdot {a^{\prime }}_{k}^{b}-{\left[\begin{array}{ccc} 0 & 0 & g \end{array}\right]}^{T}$$

(22) $$v_{k|k-1}=v_{k-1|k-1}+{\overset{⌣}{a}}_{k}\cdot \Delta t$$

(23) $$r_{k|k-1}=r_{k-1|k-1}+v_{k|k-1}\cdot \Delta t$$

where ${a^{\prime }}_{k}^{b}={\left[f_{x},\;f_{y},\;f_{z}\right]}^{T}$ is the accelerometer’s output value after a compensation filter.

### 3.2. Position Estimation Filter Design

**Figure 7:**
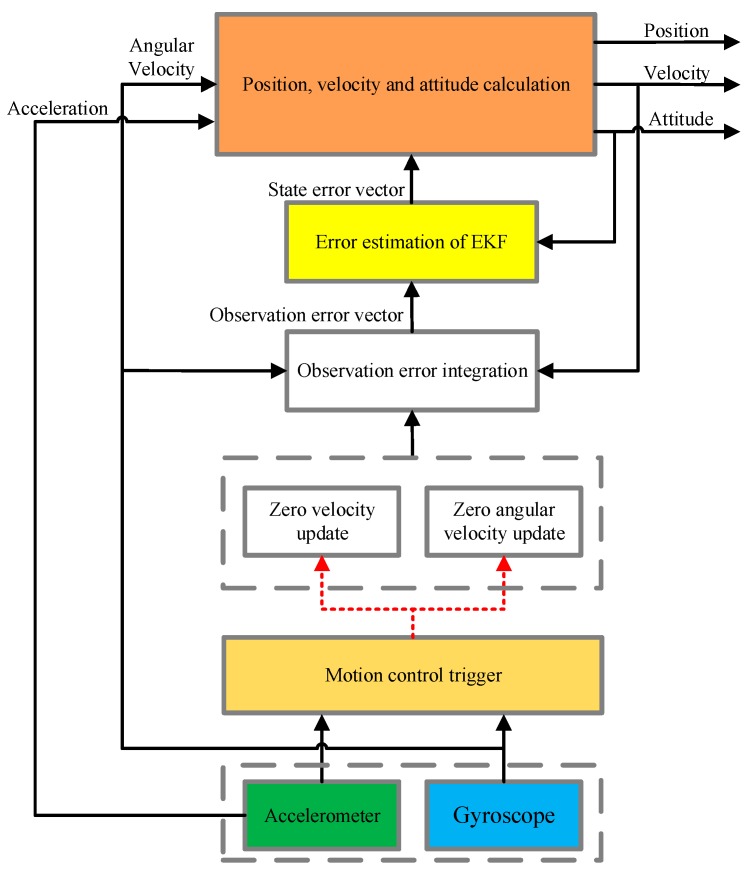
Flow diagram of the inertial navigation.

At the end of each movement cycle, the snake robot uses the controller to perform zero-velocity control, directing the robot to be static in milliseconds. It corrects the navigation error in the static moment. While the snake robot is static for a short time, its velocity and angular velocity are zero. According to the state characteristics, the mechanical arrangement design algorithm is shown in Figure 7. Using the accelerometer and gyroscope to acquire the acceleration and angular velocity information of the snake robot, the momentary stationary state of the snake robot can be obtained by analyzing the periodic data. The stationary signal is used to drive the Kalman filter to integrate its own strapdown solution. To achieve the final accurate speed, position and attitude information output. In the arrangement, the most important aspect is its core filter design [28].

**Figure 8:**
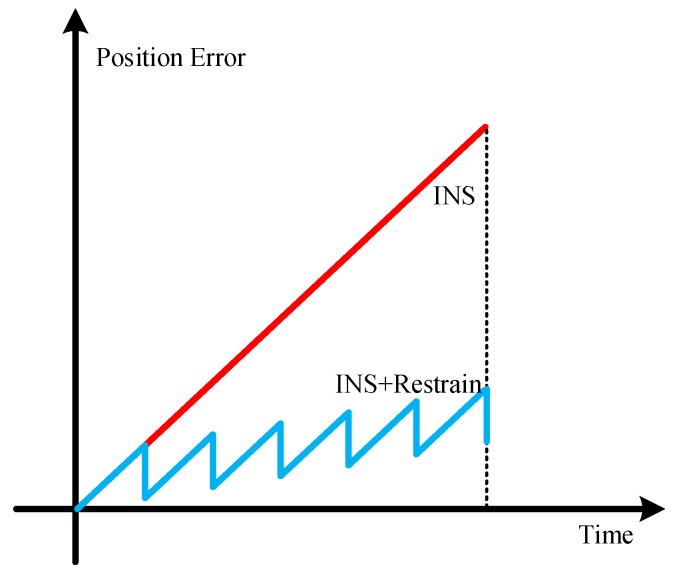
Diagram of the restraining process.

The MEMS inertial measurement unit is known to spread gradually over time, especially for a snake robot that is rapidly changing in a small cycle using the MEMS strapdown method to measure the speed, posture and position information. However, if, in a short period of time, the integral error is eliminated in a regular time, then the operation information of the snake robot can be effectively measured within a certain scope; the constraint process schematic is shown in Figure 8 [29]. If only the traditional inertial navigation solution is used, the position error will increase with the accumulation of time. Because the snake robot has the characteristics of periodic motion, we zero the speed and angular velocity to carry on the error restraint, which greatly reduces the position error.

#### 3.2.1. Establish the State Equation

According to the error propagation characteristics shown in Equation (14), build its state equation. First, choose its state variables as follows:

(24) $$X_{k}={\left[\begin{array}{ccccc} \delta \varphi_{k} & \delta \omega_{k}^{b} & \delta r_{k} & \delta v_{k} & \delta a_{k}^{b} \end{array}\right]}^{T}$$

to obtain:

(25) $$\left\{ \begin{array}{l} X_{k+1}=f\left(X_{k}\right)+G_{k}W_{k}\\ Z_{k+1}=H_{k}X_{k}+V_{k} \end{array}\right.$$

where $W_{k}$ is the system process noise matrix given by $W_{k}=[\begin{array}{cc} -C_{b}^{n}\omega^{b} & -C_{b}^{n}a^{b} \end{array}]$. $G_{k}$ is the corresponding coefficient of the noise matrix. $Z_{k}$ is the observed quantity, $H_{k}$ is the observation matrix, and $V_{k}$ is the observation noise matrix. The $f\left(X_{k}\right)$’s specific definition is as follows:

(26) $$f\left(X_{k}\right)=\left\{\begin{array}{c} \delta \varphi_{k}+C_{b}^{n}\cdot \delta \omega_{k}^{b}\cdot \Delta t\\ \delta \omega_{k}^{b}\\ \delta r_{k}+\delta v_{k}\cdot \Delta t\\ \delta v_{k}+C_{b}^{n}\cdot \delta a_{k}^{b}\cdot \Delta t\\ \delta a_{k}^{b} \end{array}\right\}$$

#### 3.2.2. Extended Kalman Filter

EKF is representative of the traditional nonlinear estimation. The basic approach is to perform partial linearization for the nonlinear state function and measurement function according to the first-order Taylor polynomial expansion and then apply the linear system Kalman filtering equation. According to this approach, we obtain:

(27) $$\Phi_{k}=\frac{\partial f\left(X_{k}\right)}{\partial X_{k}}=\left[\begin{array}{ccccc} {Ι}_{3\times 3} & \Delta t\cdot C_{b_{k|k-1}}^{n} & 0 & 0 & 0\\ 0 & {Ι}_{3\times 3} & 0 & 0 & 0\\ 0 & 0 & {Ι}_{3\times 3} & \Delta t\cdot {Ι}_{3\times 3} & 0\\ -\Delta t\cdot S(a^{\prime }{}_{k}^{n}) & 0 & 0 & {Ι}_{3\times 3} & \Delta t\cdot C_{b_{k|k-1}}^{n}\\ 0 & 0 & 0 & 0 & {Ι}_{3\times 3} \end{array}\right]$$

According to state error vector matrix, the linearization state transfer model is given as below:

(28) $$\delta x_{k|k-1}=\Phi_{k}\delta x_{k-1|k-1}+w_{k-1}$$

where $\delta x_{k|k-1}$ is the predicted state error; $\delta x_{k-1|k-1}$ is the state error after filtering at time *k* − 1; $w_{k-1}$ is process noise and can be expressed as a collaborators variance matrix:

(29) $$Q_{K}=E(w_{k}w_{k}^{T})$$

where $C_{b_{k|k-1}}^{n}$ is the transfer matrix; Δt is the sampling time; $S(a^{\prime }{}_{k}^{n})$ is a skew-symmetric matrix of the acceleration, which is used to estimate the pitch angle and roll angle of the transducer and is given by:

(30) $$S(a^{\prime }{}_{k}^{n})=\left[\begin{array}{ccc} 0 & -a_{zk} & a_{yk}\\ a_{zk} & 0 & -a_{xk}\\ -a_{yk} & a_{xk} & 0 \end{array}\right]$$

where $a^{\prime }{}_{k}^{n}$ is the acceleration value under the navigation system. The specific expression is:

(31) $$a^{\prime }{}_{k}^{n}=C_{b_{k|k-1}}^{n}\cdot a^{\prime }{}_{k}^{b}=(a_{xk},a_{yk},a_{zk})$$

The measurement model is as follows:

(32) $$z_{k}=H\delta x_{k|k}+v_{k}$$

where **z***_k_* is the observation matrix; **H** is the measurement matrix; **v***_k_* is the measurement noise, and its covariance matrix is expressed as:

(33) $$R_{k}=E\left(v_{k}v_{k}{}^{T}\right)$$

The state update equation is:

(34) $$\delta x_{k|k}=\delta x_{k|k-1}+K_{k}\cdot \left[m_{k}-H\delta x_{k|k-1}\right]$$

where **K***_k_* is the Kalman gain, **m***_k_* is a 9D measurement matrix at zero velocity, including three errors at zero velocity, three errors at zero angular velocity and three errors on the attitude angle. **K***_k_* is expressed as:

(35) $$K_{k}=P_{k|k-1}H^{T}{\left(HP_{k|k-1}H^{T}+R_{k}\right)}^{-1}$$

where **P***_k_*_|*k*−1_ is the estimate of the covariance matrix, and its expression is calculated according to the information of time *k* − 1 and is given as follows:

(36) $$P_{k|k-1}=\Phi_{k-1}P_{k-1|k-1}\Phi_{k-1}^{T}+Q_{k-1}$$

Last, update the covariance matrix:

(37) $$P_{k|k}=\left(I_{15\times 15}-K_{k}H\right)P_{k|k-1}{\left(I_{15\times 15}-K_{k}H\right)}^{T}+R_{k}$$

Repeating the above steps, the conventional EKF function of the fusion the snake robot’s motion characteristics can be realized.

### 3.3. Analysis of the Trigger Correction Mechanism under the Restriction of Snake Robot Behavior

#### 3.3.1. Velocity-Assisted Correction of the Snake Robot’s Movement

Because of the sensor’s measurement error, noise and algorithm error, the calculated speed value is not zero at speed zero. Speed-assisted correction in the design just uses this principle, which is referred to as zero-speed correction, or Zero-Velocity-Update (ZUPT). When the static snake robot is detected, obtain the error of the calculated velocity value by performing coordinate transformation and integral operation on the accelerometer’s output. The error is the observation value of the filter. During the static moment, the velocity output error is as follows:

(38) $$\Delta v_{k}=v_{k}-{\left[\begin{array}{ccc} 0 & 0 & 0 \end{array}\right]}^{T}$$

ZUPT’s corresponding observed value and the observation matrix is as follows:

(39) $$\begin{array}{l} Z_{k}={\left[\Delta v_{k}\right]}^{T}={\left[v_{k}\right]}^{T}-{\left[\begin{array}{ccc} 0 & 0 & 0 \end{array}\right]}^{T}\\ H_{k}=\left[\begin{array}{ccccc} O_{3\times 3} & O_{3\times 3} & O_{3\times 3} & I_{3\times 3} & O_{3\times 3} \end{array}\right] \end{array}$$

#### 3.3.2. Velocity-Assisted Correction and Fusion Angular Velocity-Assisted Correction

Similar to the velocity-assisted correction principle, when the snake robot is detected at the static moment in the serpentine cycle, the output angular velocity is zero, in theory. As a result of the sensor’s measurement error, noise and algorithm error, the calculated angular velocity value is not zero. Zero-presents-rate-update (ZARU) refers to the Zero angular velocity correction, i.e., the error of the gyroscope’s angular velocity output is used as the observed quantity for the filter when the snake robot motion model detects the IMU to be static.

ZARU’s corresponding observed value and the observation matrix is as follows:

(40) $$\begin{array}{l} Z_{k}={\left[\Delta w_{k}\right]}^{T}={\left[w_{k}\right]}^{T}-{\left[\begin{array}{ccc} 0 & 0 & 0 \end{array}\right]}^{T}\\ {Η}_{k}=\left[\begin{array}{ccccc} O_{3\times 3} & I_{3\times 3} & O_{3\times 3} & O_{3\times 3} & O_{3\times 3} \end{array}\right] \end{array}$$

To achieve a higher precision, using ZUPT and ZARU at the same time, the corresponding observed value and the observation matrix is as follows:

(41) $$\begin{array}{l} Z_{k}={\left[\begin{array}{cc} \Delta v_{k} & \Delta w_{k} \end{array}\right]}^{T}={\left[\begin{array}{cc} v_{k} & w_{k} \end{array}\right]}^{T}\\ H_{k}=\left[\begin{array}{l} \begin{array}{ccccc} O_{3\times 3} & O_{3\times 3} & O_{3\times 3} & I_{3\times 3} & O_{3\times 3} \end{array}\\ \begin{array}{ccccc} O_{3\times 3} & I_{3\times 3} & O_{3\times 3} & O_{3\times 3} & O_{3\times 3} \end{array} \end{array}\right] \end{array}$$

**Figure 9:**
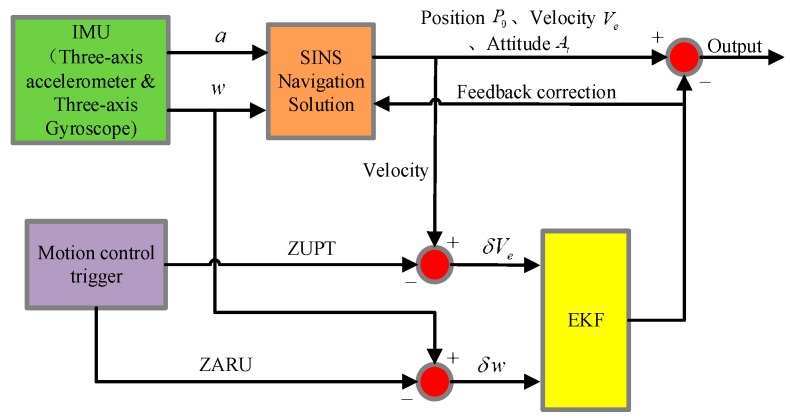
Flow diagram of the correction solution.

The process of the method is shown in Figure 9: This figure details the navigation solution process for zero speed and zero angular velocity correction. Each periodic static motion of the snake robot is triggered by a motion controller, and then the Kalman filter observations are corrected at this moment to correct the attitude, velocity and position information of the navigation solution and improve the navigation accuracy.

## 4. Experiment

### 4.1. Prototype Development

**Figure 10:**
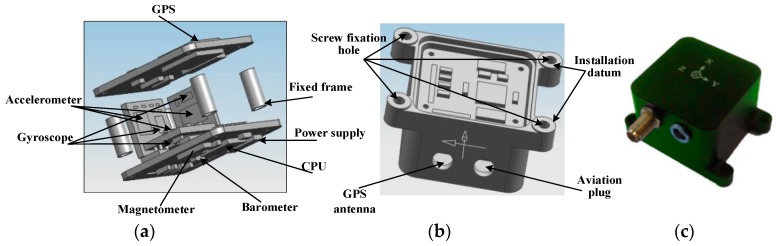
IMU and internal structure. (**a**) Exploded views of the IMU; (**b**) Assembly views of the IMU; (**c**) Overall views of the IMU.

The snake robot consists of a total of 6 joints, and 5 sets of servos. In each joint, there is a Ni-MH battery pack and a slave controller. At each link, two Futaba S9157 servos are mounted orthogonally, each servo with a speed of 0.14 s/60° @ 6.0 V and a torque of 30.6 kg·cm @ 6.0 V. The snake robot’s head structure mainly includes an IMU system (Figure 10) and master controller circuits. Slave controller and the IMU system communicate with the master controller through the RS-422 bus. The sensor head also integrates a high-resolution camera that can provide a video image for remote operation personnel, LED lighting for the camera system, IR sensors for obstacle avoidance and a Ni-MH battery pack that is used as a power supply for the entire system.

#### 4.1.1. Design Process

**Figure 11:**
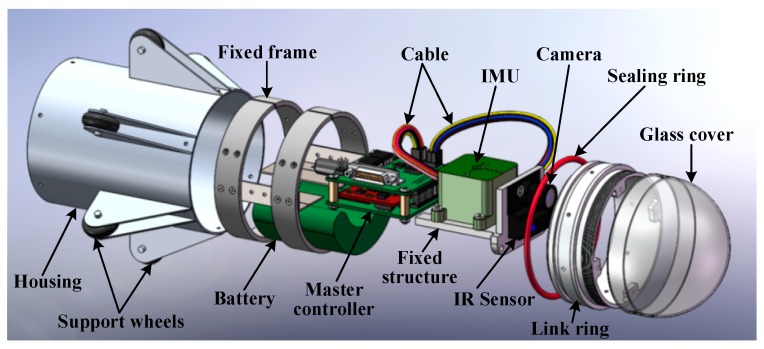
Exploded views of the sensor head.

**Figure 12:**
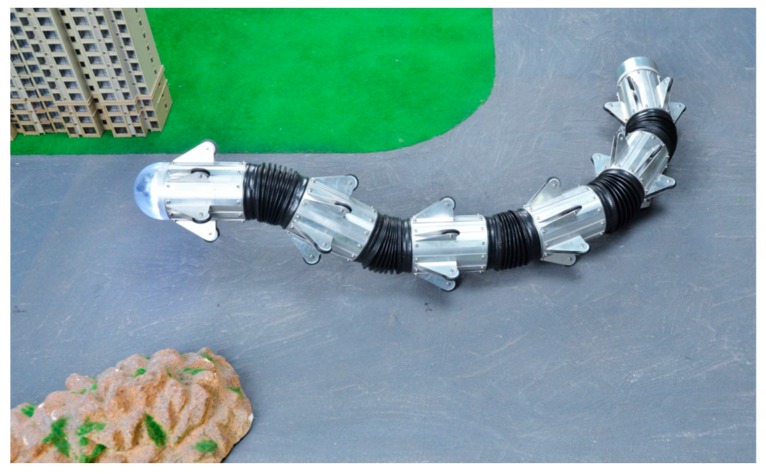
Snake robot body.

3D model printing technology is used in the iterative design of the snake robot. It can reduce the design cycle and cost. Ultimately, the snake robot’s shell and body use aluminum alloy to maintain the structural strength and decrease the overall weight of the snake robot to improve its movement flexibility. The robot parts all use socket head cap screw fasteners, which is convenient for disassembly. The internal circuit and battery of the joint are fixed on the Teflon elastic double O-type frame to ensure its shake-resistance and stability. In each of the joints of the external structure, an O-type rubber ring is used to prevent water and other debris from entering the robot. In the joints of the movement mechanism, a steel wire-reinforced canvas bellows material is used to ensure movement flexibility, as well as waterproofing and wear-resistance capability. The details of the connections between the structures are shown in Figure 11. The snake robot’s head is a specially designed scratch-resistant glass. It improves the robot’s environmental compatibility, providing features such as anti-scratch and wear-resistance abilities, which do not decrease the camera transmission efficiency and maintains acceptable distortion. The final installed and integrated snake robot is shown in Figure 12.

#### 4.1.2. IMU System Sensor

The IMU system mainly comprises three high-precision single-axis accelerometers (Model: ADXL103, Analog Devices, Inc., Norwood, MA, USA) and gyroscopes (Model: ADXRS642, Analog Devices, Inc., Norwood, MA, USA), GPS, a barometer, a three-axis magnetometer, a 32-bit ARM kernel main controller chip, a 16-bit AD data-acquisition circuit, and a secondary regulated power supply. The main technical indicators are as shown in Table 1. The main controller can measure the real-time sensor data, position, velocity and attitude information of the output carrier through EKF (note: in this paper, the GPS, barometer and magnetometer are turned off to study the inertial navigation precision). The 16-bit AD data-acquisition circuit can ensure the accuracy of the acquisition data from the sensor. The secondary regulated power supply can ensure a stable power supply for the system. The IMU system fixes the snake robot’s head structure through a specially designed frame to ensure its rigid connection and installation accuracy. The IMU measurement data can also be sent to the PC through the main controller at the same time via the ZigBee sensor. We also provide a variety of control mode designs for the system in order to adapt to different test scenarios, including automatic control, infrared, Bluetooth, PC serial port control and so on.

Finally, the host controller PCB includes all communication and power supply circuits and integrates the temperature and humidity sensors and current monitoring module to monitor the environment information and running status of the power supply system.

### 4.2. Experimental Verification

**Figure 13:**
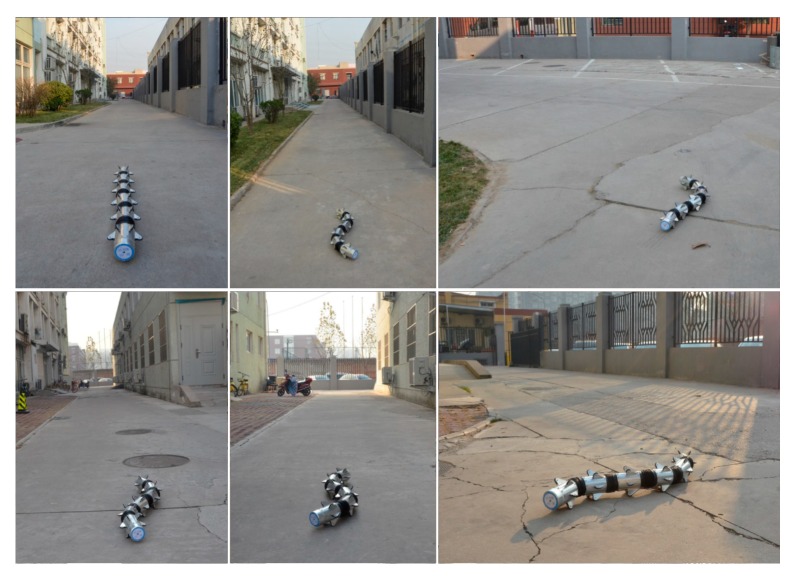
Outdoor experiment.

To verify the feasibility and precision of the above algorithm, considering the snake robot’s running state in the actual use, we designed a linear motion, turn motion, turn-back motion and circular motion. The test scenario is on a rough cement road, as shown in Figure 13. The actual trajectory of the robot was obtained by tagging the snake robot’s motion track points during the process.

Over the entire test process, the movement information from IMU was sent to the host computer for real-time monitoring and was collected via ZigBee.

#### 4.2.1. Linear Motion Test

Before the linear motion, the snake robot’s master controller set a target point, which was 15 m away from the origin on the host computer, and then set the snake robot to static for 200 ms at the end of each movement cycle. The host computer collects real-time sensor data and monitors the running status of the snake robot using the zero speed and zero angular velocity constraints, respectively, to verify the algorithm’s feasibility and accuracy.

**Figure 14:**
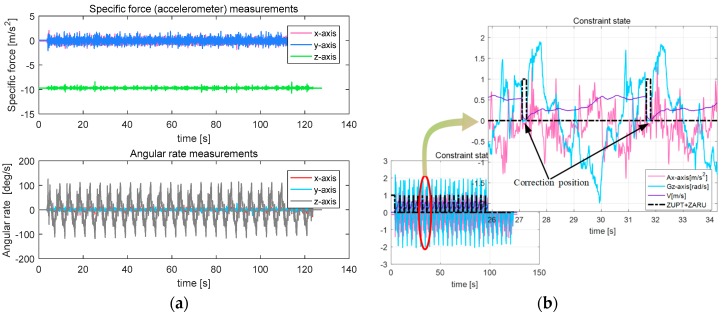

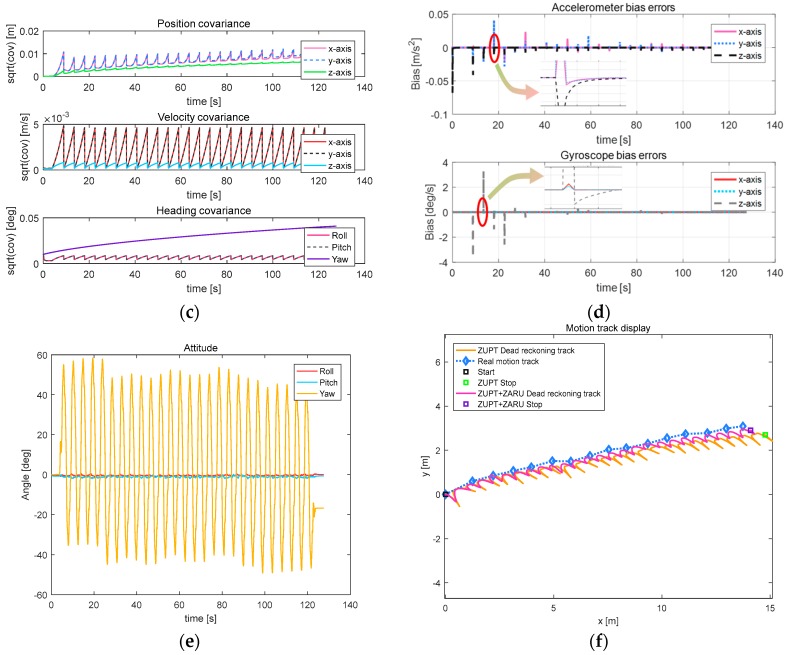
Parameters related to linear motion under velocity and angular velocity constraints. (**a**) Output result of the MEMS IMU; (**b**) Constraint state; (**c**) Result of the covariance; (**d**) Bias errors; (**e**) Attitude of the snake robot; (**f**) Moving path of the snake robot.

Figure 14a (top) shows the original three-axis acceleration values collected by the sensor during the linear motion of the snake robot. Figure 14a (bottom) shows the original values of the three-axis angular velocity. The abscissa of the two graphs is the time-axis, the unit is second. Figure 14a shows that, in the process of snake robot motion, its acceleration and angular velocity are cyclically changed. The accelerometer *x*-axis and *y*-axis represent the snake robot’s motion speed change state. The data noise is higher because of the rough uneven ground. The *z*-axis is mainly for Earth’s gravity. The gyroscope’s *x*-axis and *y*-axis represent the snake robot’s pitch and roll motion. Because there is no pitch and roll motion in serpentine movement, the *x*-axis and *y*-axis angular rate value is small. The *z*-axis is a sensitive element of the snake robot in serpentine angle motion, as it is sufficiently sensitive to the serpentine movement periodicity of the robot.

Figure 14b shows the constraint points for each cycle in the snake robot’s linear motion. The enlarged image is a magnified view of two cycles inside the red circle. Where the red curve is the *x*-axis acceleration value, the blue curve is the *z*-axis angular velocity value (shown as a gray curve in Figure 14a (bottom)), and the purple curve is the robot velocity value. Black dotted lines indicate the constraints (represented by 0 and 1), and the positions indicated by the arrows are the constraint points for each cycle. As can be seen from the enlarged image in Figure 14b, the snake robot is static for 200 ms in each cycle. During this period, zero speed and zero angular velocity correction constraints are triggered. As shown by the black dotted lines in Figure 14b, the algorithm can accurately detect every zero-velocity moment and correct in each cycle.

The images of Figure 14c (top, middle and bottom) show the covariance of position, velocity and attitude, respectively. Covariance is an important index for verifying whether the Kalman filter is normal. It can be seen from the figure that the covariances of position and attitude angle gradually become larger, and the covariance of velocity basically changes periodically. This is where the position and attitude of the robot keep accumulating changes, and the velocity error will be corrected every time the robot’s zero velocity point is detected. The rapid convergence of covariance per cycle indicates that the Kalman filter is working properly and can effectively estimate the systematic error.

The images of Figure 14d (top and bottom) show the zero errors of the accelerometer and the gyroscope observed by the Kalman filter, respectively. It can be seen from the figure that the zero error can be estimated and converged at each cycle.

The pitch and roll axis attitude angle is found to exhibit basic fluctuations near zero, with the heading axis attitude angle periodically changing based on the robot’s serpentine movement, as shown in Figure 14e. The resulting trajectory curve is shown in Figure 14f; the orange curve is the motion curve under the velocity restriction, with a horizontal error of 0.85 m. The red curve is the motion curve under velocity and angular velocity restriction, with a horizontal error of 0.41 m. The horizontal error is 6.07% and 2.92% of TTD, respectively (the results of the other three tests are: 6.83% and 3.65%, 5.94% and 2.95%, and 6.36% and 3.27%). From the above data, the horizontal error precision under velocity and angular velocity restriction is increased by 3% compared to that under velocity restriction only. Because of the length restrictions of this paper, we only consider the situation under velocity and angular velocity restrictions in the next tests.

#### 4.2.2. Turn Motion Test

In the actual use of a snake robot, the turn motion is the most common movement scenario. After the passing the linear motion test, the next test is to set two target points that are on the vertical direction relative to the start point by the host computer to examine the feasibility of the algorithm in larger heading deviation.

**Figure 15:**
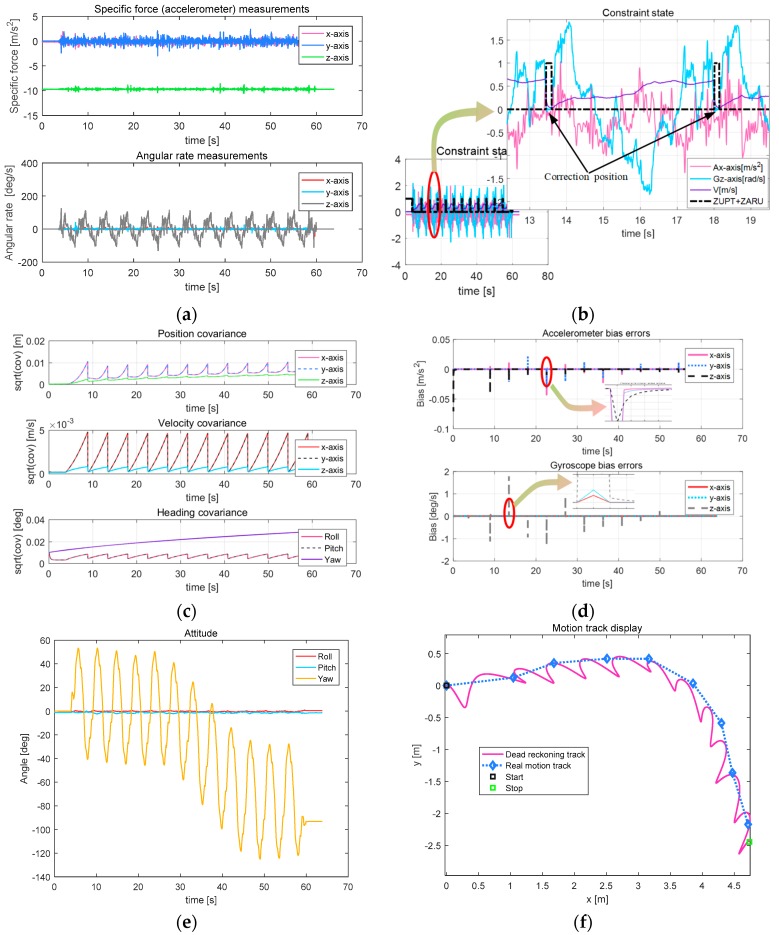
Parameters related to turn motion. (**a**) Output result of the MEMS IMU; (**b**) Constraint state; (**c**) Result of the covariance; (**d**) Bias errors; (**e**) Attitude of the snake robot; (**f**) Moving path of the snake robot.

In Figure 15a–d, the snake robot is performing the serpentine movement in a fixed period. Even for the large turning radius, the accelerometer data changes little. The cyclicality of constraints, covariance of position, velocity and attitude, bias error convergence of accelerometer and gyroscope are consistent with those of linear motion.

Figure 15e shows that the robot’s heading attitude angle has changed 90 degrees, and the other two axes are still approximately zero. From the resulting trajectory in Figure 15f, the actual walking distance is 6.5 m, the horizontal error is 0.28 m, 4.31% of TTD (the results of the other three tests are: 4.25%, 4.97%, 3.84%).

#### 4.2.3. Turn-Back Motion Test

In addition to the straight line and turn movement, a typical motion of a robot is to return to the origin in the mission process. After passing the tests of the algorithm in a straight line and turning movements, the next test is to reset the two target points by the host computer: the endpoint to go to (15 m away from the start point) and the endpoint to return to. Further investigation on the algorithm’s feasibility for larger heading turn states and for the return path of the snake robot is shown below.

**Figure 16:**
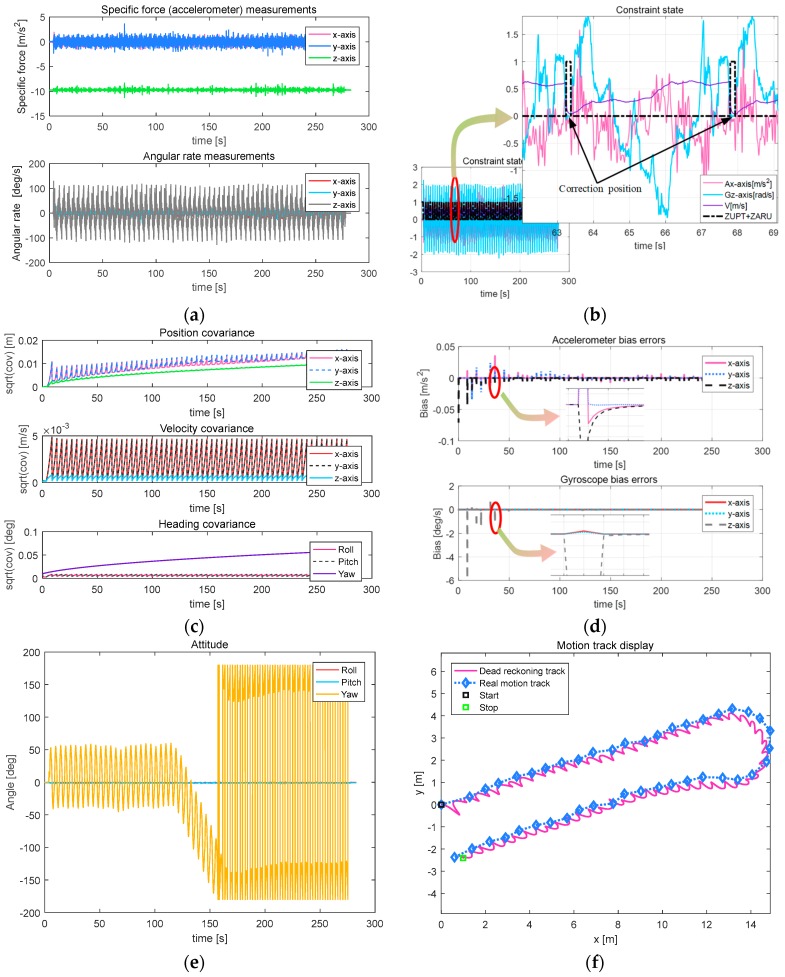
Parameters related to turn-back motion. (**a**) Output result of the MEMS IMU; (**b**) Constraint state; (**c**) Result of the covariance; (**d**) Bias errors; (**e**) Attitude of the snake robot; (**f**) Moving path of the snake robot.

Figure 16a–d shows that, because the turn-back movement obviously has a long range and more serpentine cycles, the convergence of data, including accelerometer and gyroscope data, constraint cyclicality, the covariance of position, velocity and attitude, and bias errors of the accelerometer and gyroscope, are consistent with those of both linear and right-angle turn movements.

Figure 16e shows that the robot’s heading attitude angle has changed 180 degrees because of the turn-back movement, with the other two axes remaining approximately zero. From the resulting trajectory in Figure 16f, the actual walking distance is 31.6 m, the horizontal error is 0.58 m, 1.84% of TTD (The results of the other three tests are: 2.37%, 3.01%, 2.18%).

#### 4.2.4. Circular Motion Test

**Figure 17:**
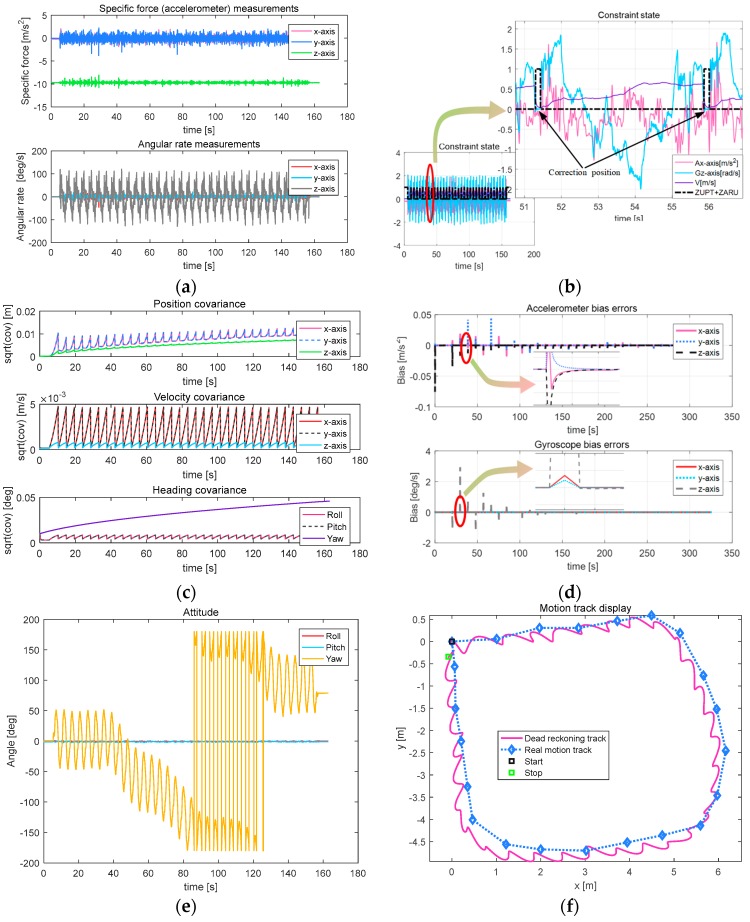
Parameters related to circular motion. (**a**) Output result of the MEMS IMU; (**b**) Constraint state; (**c**) Result of the covariance; (**d**) Bias errors; (**e**) Attitude of the snake robot; (**f**) Moving path of the snake robot.

After passing the tests of the algorithm in straight line, turn and turn-back movements, the last test is to design a type of circular motion to evaluate the algorithm’s accuracy in returning to origin after many turns and linear motions. Because of the limitations of space, four target points (5 m × 5 m rectangular) are reset by the host computer; the motion curve is shown in Figure 17f.

Figure 17a–d shows that the convergence of data, including accelerometer and gyroscope data, constraint cyclicality, the covariance of position, velocity and attitude, and bias errors of the accelerometer and gyroscope, are consistent with those of linear, turn and turn-back movements.

Figure 17e,f shows that the snake robot’s heading axis changed three times in all, basically returning to origin, eventually; however, the heading has changed 90 degrees from the starting moment. The other two axes are still approximately zero. Figure 17b,f shows that the algorithm can effectively and accurately detect the zero-velocity moment and constrain the divergent serious inertial navigation positioning data. It can achieve a trajectory curve that is quite smooth and has highly repetitive start and end points. The simulation trajectory is consistent with the actual trajectory. With respect to the resulting trajectory curve, its horizontal error is 0.35 m, the actual walking distance is 19.4 m, 1.80% of TTD (the results of the other three tests are: 2.52%, 2.67%, 1.75%).

In summary, its location precision is within 5% of TTD in regular motion types using the proposed navigation method based on the characteristics of the snake robot motion constraints. This is able to satisfy the requirement of autonomous navigation and positioning for the snake robot in traditional applications in short distance running situations.

## 5. Conclusions

This paper described a proposed autonomous navigation method based on the characteristics of a snake robot’s motion constraints. The proposed method realized the snake robot’s autonomous navigation and positioning with non-nodes and non-assistant using the installation of IMU on the robot. By studying the motion characteristics of the snake robot, it establishes its kinematics model, analyzes the movement constraint characteristics and movement error propagation characteristics, explores the snake robot navigation arrangement, proposes constraint criteria and fixed relationships, and satisfies the zero-state constraint with the motion feature and control modes of the snake robot. Finally, it studies the snake robot’s EKF position estimation method under motion characteristics restriction, realizing the robot’s autonomous navigation positioning. The tests of linear, turn, turn-back, and circular motion with the self-developed snake robot show that its comprehensive location accuracy was less than 5% of TDD, and proved that in short run situations, the method can meet the requirements of autonomous navigation and positioning for snake robots.

Although this method has been applied to the field of vehicle and pedestrian navigation for a long time, it has not been applied to snake robots because of the uniqueness of the movement characteristics of snake robots. The novelty of this paper is in applying this technology to the snake robot’s navigation by studying the motion model of the snake robot and to study the feasibility of its short-distance navigation. The future work is to continue improving the key technology of long-distance long-time autonomous navigation, and to apply this work to the robot SLAM system to improve the accuracy of positioning and mapping. Finally, a snake robot with high-precision navigation can be widely used in disaster rescue, underwater survey, industrial testing and other special environments that traditional robots or humans cannot enter.

## Figures and Tables

**Table 1:** Main technical indicators of the MEMS IMU.

	Specifications	Index Value
Accelerometer	Range	±1.7 g
	Bias Instability	25 mg
	Non-linearity	<0.2%
	Velocity random walk	<0.75 m/s/h^1/2^
	Bandwidth	2500 Hz
Gyroscope	Range	±300°/s
	Bias Instability	20°/h
	Non-linearity	<0.1%
	Angle random walk	<0.02°/s^1/2^
	Bandwidth	2000 Hz
Systems	Update Time	5 ms
	Power	0.15 A @ 5 Vdc
